# Zirconium dioxide implants as an alternative to titanium: A systematic review

**DOI:** 10.4317/jced.58063

**Published:** 2021-05-01

**Authors:** Ivana Comisso, Santiago Arias-Herrera, Saurabh Gupta

**Affiliations:** 1 Universidad Europea de Valencia. Faculty of Health Sciences. Department of Dentistry; 2 Clinical and Applied in Implant-Prosthetics (ICAI) Research Group, Universidad Europea de Valencia. Faculty of Health Sciences. Department of Dentistry; 3 Zirconia Implant Research Group, Silver Spring, MD, 20910, USA; 4 Master Dental Science, Universitat Jaume I, Castellón, Spain

## Abstract

**Background:**

This systematic review evaluates the available scientific literature to demonstrate the aesthetic and clinical benefits and to determine the survival and the success of zirconium dioxide implants concerning titanium implants.

**Material and Methods:**

The electronic databases were searched until January 2020. Outcome measures were pink aesthetic score (PAS), white aesthetic score (WAS), bleeding on probing (BOP) and probing depth (PD). The addressed PICO question was: In partially edentulous patients in the upper-anterior sector, do zirconium dioxide implants provide aesthetic benefits over conventional titanium implants?

**Results:**

In the 15 articles included after the screening were evaluated respectively, the pink aesthetic score, white aesthetics score, the peri-implant, and crown index, bleeding on probing, plaque index, probing pocket depth, radiographic bone loss, papilla height, and survival and success rate. The results show how the aesthetic and clinical benefits and the survival and success rate of zirconium dioxide implants are, in general, better than titanium implants.

**Conclusions:**

Despite the optimal aesthetics, clinical and survival results obtained in the review, more studies are needed to confirm these data.

** Key words:**Ceramic dental implants, zirconia implants, esthetics, zirconium dioxide and zirconia.

## Introduction

In recent years, the treatment options and modalities to achieve optimal functional and aesthetic results in dental implants have progressively changed and have improved the quality of life of many patients. Titanium is the gold standard and a reliable implant material in dentistry, but it’s greyish colour can lead to aesthetic problems. In some situations, there may be a deficiency of soft tissue above the implant level at the time of definitive restoration, or this may occur after the loss of marginal bone with mild tissue recession; in such situations, there is an unsightly display of the metal components (1).

Therefore, the research has focused on the search for material for the implant that has the same color as of the tooth, that improves its aesthetic appearance and, at the same time, that is highly biocompatible and able to withstand the occlusal forces in the oral cavity (2).

Zirconium dioxide seems to be suitable as a material of choice in the preparation of dental implants because of their tooth-like color, their mechanical properties, and therefore their biocompatibility. The use of zirconium dioxide implants avoids the phenomenon of loss of apical bone and gingival recession that normally occurs in metal implants, and also accesses the request of many patients to be “metal-free” (1-4).

At present, there are limited studies that evaluate the aesthetic benefits of using zirconium dioxide implants as an alternative to titanium implants in esthetics areas. Macro/micro design, peri-implant tissue response, surgical protocols, as well as, the comparison between both types of implants induces conflicting results in the literature studies. It is necessary, therefore, to conduct a systematic review of published trials to synthesize, and to clarify the published literature so that it can serve as a basis for future studies.

Therefore, this systematic review aims to evaluate the aesthetic benefits of using zirconium dioxide implants as an alternative to titanium implants.

## Material and Methods

-Protocol and registration

The review was registered in PROSPERO, an International Prospective Register of Systematic Reviews under number. The protocol is accessible through the following link. The Preferred Reporting Items for Systematic Review and Meta-Analysis (PRISMA) guidelines were followed to perform this systematic review (5).

-Focused question

The following focus question was employed according to the population, intervention, comparison, and outcome study design. In partially edentulous patients in the upper-anterior sector (P), do zirconium dioxide implants (I) provide aesthetic benefits (O) over conventional titanium implants (C)?

-Selection criteria

All studies selected for the systematic review had to follow the following inclusion criteria. Regarding the type of research, they had to be experimental, epidemiological studies, quasi-experimental or analytical studies (cohort studies) and intervention (clinical trial, field trial, community trial) in humans.; the sample should be made up of adult subjects partially edentulous in the upper-anterior sector. The experimental group should have been treated with zirconium dioxide implants while the control group with titanium implants. The studies also had to have a minimum follow-up of 12 months and take into account at least the variables of pink aesthetic score, white aesthetic score, bleeding on probing, and probing depth. Animal studies and articles published before 2010, not available in English, were excluded; case reports, case series, pilot studies, narrative literature reviews, and letters to the editor were also excluded.

-Search strategy

The authors performed initial electronic research in MEDLINE via Pub-Med and Cochrane Central Register of Controlled Trials until January 2020. The literature search was conducted using the combinations of the following Medical Subject Heading (MeSH) and text words: (partially edentulous OR aesthetic sector OR upper maxilla OR upper anterior maxilla) AND (zirconia dental implants OR zirconium oxide OR dental implants) AND (titanium implants OR dental implants) AND (gingival index OR bleeding on probing OR peri-implant bleeding OR peri-implant health index OR peri-implant probing depth OR pink aesthetic score OR white aesthetic score). A manual search of the reference list was used to identify additional articles.Additional relevant articles were searched manually from the reference lists of full text not to exclude any publication of interest.

-Screening methods and data abstraction

Two reviewers (IC and PS) in duplicate and independently performed the systematic review search. Once the duplicate studies between the different databases had been removed, titles and abstracts of all identified studies were screened for eligibility. During this phase, the articles were excluded because they were published before the established date (2010) or because they did not fit the study topic. The full text of all the studies selected in the first phase was read, and the inclusion and exclusion criteria were applied. Any disagreement was resolved with the discussion between both reviewers until consensus was reached or through arbitration by a third examiner (S.A). The level of agreement was calculated using the kappa static according to the criteria of Landis and Koch (6).

Data was extracted from accepted studies, including the following details. Authors name, year of publication, country, subjects (sample size, mean and age range in years and male to female ratio), study groups, and follow-ups. In addition to the following variables: pink aesthetic score (PAS), white aesthetic score (WAS), peri-implant and crown index (PCI), bleeding on probing (BoP), plaque index (PI), probing depth (PD), radiographic bone loss (RBL), papilla height and survival and success rates.

-Risk of bias in individual studies

The risk of bias was assessed independently and in duplicate by the two authors (IC and PS) according to the Cochrane collaborations’ tool (7). Bias is evaluated as a judgment (high, low, or unclear) for individual elements from five domains: Bias arising from the randomization process; bias due to deviations from intended interventions, due to missing outcome data, the bias in the measurement of the outcome and preference in selection of the reported result. Overall, studies were considered as Low Risk of bias if the trial is judged to be at low risk of bias for all domains, Unclear if the test is decided to raise some concerns in at least one area for this result, but not to be at high risk of bias for any domain and High Risk of preference if the study is judged to be at high risk of bias in at least one area for this result or the trial is decided to have some concerns for multiple domains in a way that substantially lowers confidence in the result. The quality of studies other than RCT was assessed using the Newcastle Ottawa Scale (8). Other sources of bias were also registered and taken into account including Internal validity (inclusion criteria), external validity (randomization, concealment, patient, operator and examiner blinding, lost and abandoned and intervention treatment), statistical analysis, evaluation method, examiner calibration, data reproduction, validation of measurements, placebo and patient compliance.

-Statistical analysis

The articles were compared, and the mean values of the primary variables were directly grouped and analyzed using standardized mean difference (SMD) and 95% confidence intervals (CI). All analyses were performed with the IBM® SPSS® Statistics version 21.00 software. Statistical significance was defined for a value of *p* <0.05.

## Results

-Study selection

A total of 1109 articles were identified in the literature, electronic search (n=335), manual search (n=691) and (n=83) of the cross searches. Duplicate records between sources were removed (n=5). The rest of the studies were screened by title and abstract (n=1104). A total of 471 articles were eliminated for not being experimental, epidemiological studies, quasi-experimental or analytical studies and intervention studies in humans (n=350). Few of them were eliminated based on publication date (n=3) and the rest were excluded because they were not relevant for the objective of this review (n = 118).

**Figure 1:**
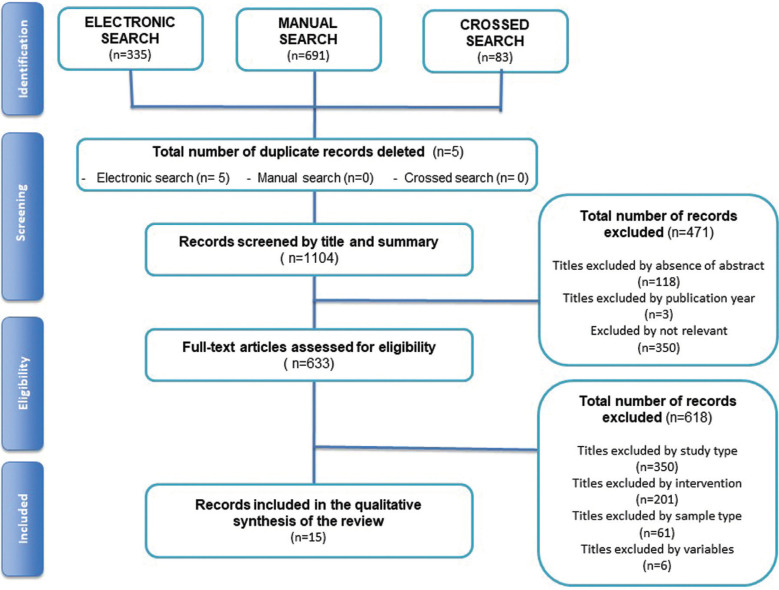
Study selection process and results of the literature search (PRISMA flow diagram).

The remaining studies were full text screened (n=633), and 618 were excluded because they did not meet the inclusion criteria. Finally, fifteen studies were included in this review (13-27). The inter-assessor agreement was good to excellent at initial screening and full-text eligibility (k =0.86 and k=0.98, respectively) (6). Figure 1 shows the flow diagram of the study selection process and results of the literature search according to PRISMA guidelines (5).

-Characteristics of included studies

All the characteristics of the studies carried out in the review are summarized in Table 1 and  Table 2. Out of fifteen selected studies, three articles were retrospective cohort studies (9,10,12), five articles were prospective cohort studies (11,13,15,16,23), two articles were retrospective case series (14,17), three articles were a prospective case series (18,30,32) and two studies were randomized clinical studies (20,22).

**Table 1:**
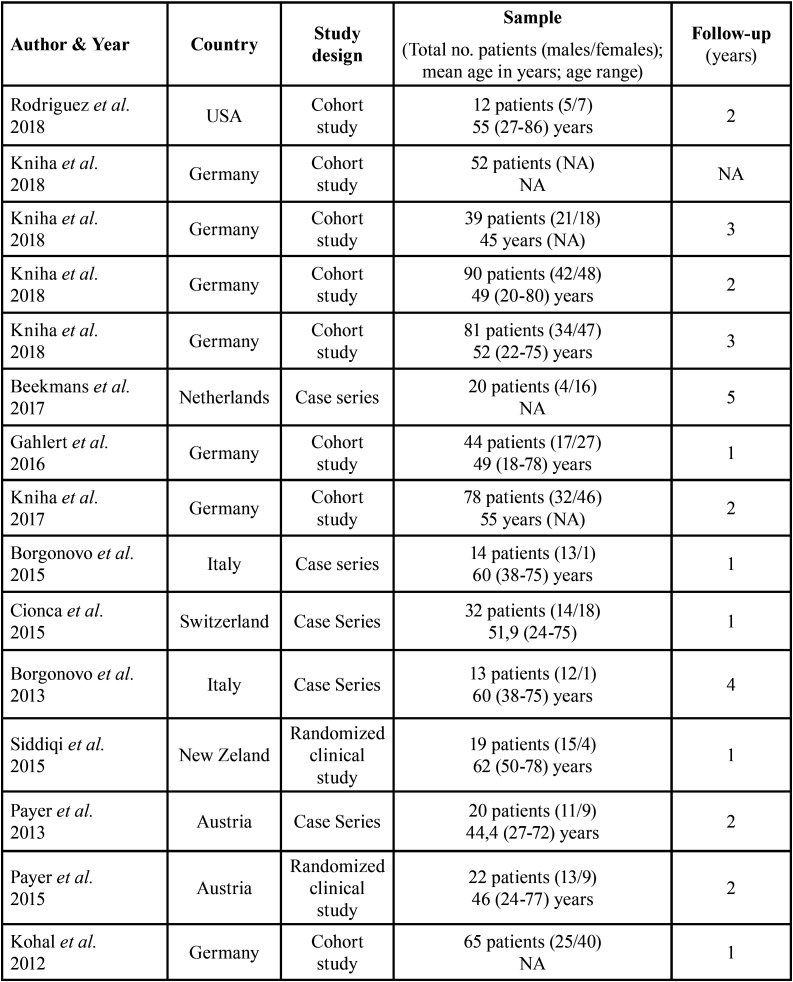
Methodology of included studies.

**Table 2:**
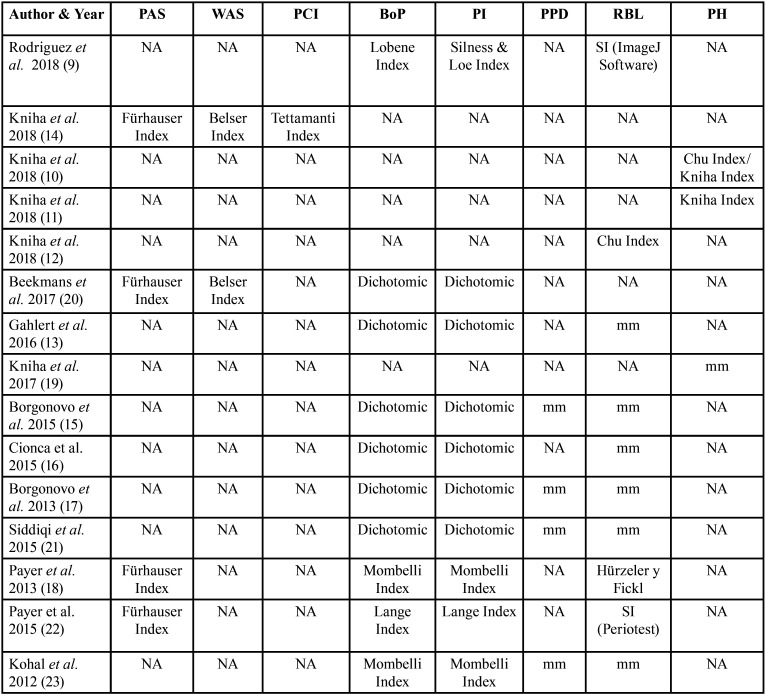
Clinical variables.

In the included studies, the number of patients ranged between 12 and 81 years (9-23). Ten studies reported that the average age of the patients was 52 years (9-18), with an age range between 18 and 86 years (9-23). The follow-up time of the articles was from 1 year to 5 years (9-23). In 13 included articles, the sex of the patients was specified (9,11-19,21-23), establishing a total of 258 men and 291 women. In two articles, the sex of the patients included in the studies is not specified (10,20). No studies define the race or ethnicity of the patients (9-23). Of the included clinical studies, three of them make a comparison between zirconium dioxide implants and titanium implants (15,20,22). At the same time, twelve articles describe only zirconium dioxide implants using them as mono-therapy (9-19,21,23). Different zirconium dioxide implants were used in these studies. There were four Straumann Bone Ceramic® articles (10-12,15,16), three articles employ the WhiteSky® implants (17,19,21), two articles employ the Yttria- implants stabilized Tetragonal Zirconia Polycrystal® (Y-TZP) (20,23), an article uses Z-Systems® implants (13), an article uses White Implants® (14), an article uses Zeramex® implants (18). Finally, in an article, they use zirconia / Ziterion vario Z® and titanium / Ziterion vario T® (22).

-Synthesis of the results

Different measures for oral hygiene (PI, BoP, PPD) and aesthetics (PAS, WAS, PCI),as well as, survival and success rates had been compared between zirconium dioxide and titanium implants. Zirconium dioxide implants have shown improved aesthetic benefits in the PAS and WAS compared to conventional titanium implants. Zirconium dioxide implants have other clinical benefits compared to conventional titanium implants, showing less plaque accumulation and, therefore, less inflammation around the peri-implant mucosa. Zirconium dioxide implants have shown a lower probing depth and an ideal papilla crown proportion with increase in papillary height. For survival and success rates not significant statistical differences were found between groups.

-Risk of bias across studies

**Figure 2:**
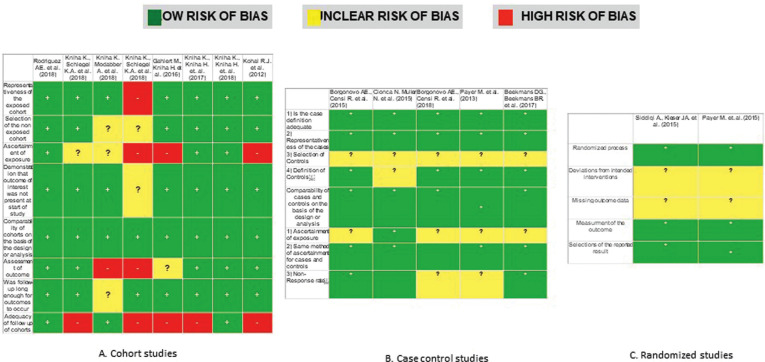
Risk of bias according to the Newcasstle-Ottawa quality assessment scale.

**Figure 3:**
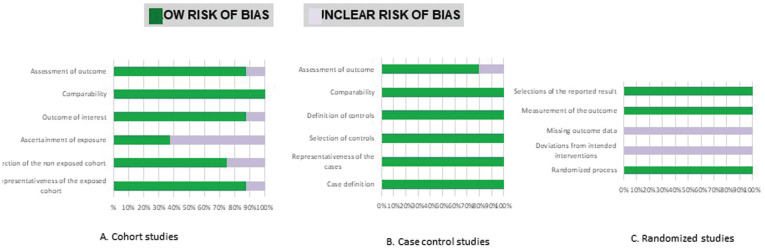
Risk of bias summary, review authors’ judgments about each risk of bias item presented as percentages across all included studies.

All included studies were evaluated (9-23), and Figure 2. summarizes this analysis. Two of the fifteen studies were classified as low risk of bias (12,22), eight had an unclear risk (12-16,19,21,23), and four of the trials had a high risk of bias (9-10,15-16). Figure 3. shows review authors’ judgments about the other risk of bias items analyzed as percentages across all included studies.

## Discussion

In this systematic review, zirconium dioxide implants were investigated. The aesthetic benefits, clinical benefits, and survival/success rate of the use of zirconium implants were evaluated as an alternative to titanium implants. However, most of the articles analyzed did not make a comparison between titanium implants and zirconium implants, but an analysis of the properties of zirconium dioxide implants. In the studies included in this systematic review, it was determined that, of the 15 included studies, only 3 of them (15,20,22) compared zirconium dioxide implants with titanium implants. Besides, the zirconium dioxide implants used in these studies were of different trademarks. However, the clinical validity criteria was a critical point, because the design of the studies was not homogeneous three articles were retrospective cohort studies (9-10,12), one article was a retrospective cohort study with examiner calibration (11), four articles were cohort studies (13,15,16,23), two articles were retrospective case series (14,17), three articles were prospective case series (18-19,21) and finally, two articles were randomized clinical studies (20,22). Also, only in one article (18) was the examiner calibration, and in all studies, the examiners were internal. The measurements were not validated, nor was the placebo formulation. Due to this heterogeneity of the studies, it is not possible to make an exhaustive comparison.

-Aesthetic benefits of zirconium dioxide implants compared to conventional titanium implants.

The pink and white aesthetic index, according to Cho *et al.*, are considered the parameters for an objective evaluation of the soft tissue aesthetics of single implants in the anterior region (24). According to Cosyn *et al.* as well as by Belser *et al.*, the acceptable values of the pink aesthetic index and the white aesthetic are> 6, the sum of the pink and white aesthetic index must be> 12, while for the peri-implant index, according to Tettamanti, > 360 points (25,26). In the study by Kniha *et al.*, the pink aesthetic index (PES) was 8.8, and the white aesthetic index (WES) was 8.6, with a total of PES + WES of 17.4, while the peri-implant index was 532.2 points (10). In general, different studies have shown that the values of pink, white aesthetics, and the peri-implant and crown index are lower for titanium implants. For example, according to a study by Belser *et al.*, the value of the pink aesthetic index for titanium implants was 7.8, the value of the white aesthetic index was 6.9, with a total PES + WES of 14.7 (26). In the study by Beekmans *et al.*, the values of the pink aesthetic index were 12.8 at baseline, and there were no changes during the reviews (14). In other studies, de Belser *et al.*, Raes *et al.* and, Cosyn *et al.* showed that the white aesthetic index values in zirconium implants vary between 6.9 and 8.2 (29,31-32).

-Clinical benefits of zirconium dioxide implants compared to conventional titanium implants.

Regarding plaque indices, in our studies, it was shown that in zirconium dioxide implants due to the lower affinity of the surface of zirconium dioxide, there was less accumulation of plaque, thus allowing a lower risk of inflammation and infections. These results are also confirmed by a study by Scarano *et al.* which demonstrated that zirconium dioxide had a lower affinity for bacterial colonization by comparing the surface area covered by bacteria after 24 hours. The results were respectively 12.1% for zirconium implants and 19.3% for titanium implants (29). Regarding the height of the papilla, according to our clinical outcomes, in general, there was an increase in the height of the papilla in zirconium dioxide implants. The literature evaluating the soft tissue interface around implants appears to favor zirconium dioxide implants over titanium implants, although more research is needed.

Regarding the height of the papilla, the results we have obtained (12) demonstrate that the papilla deficit is related to two vertical distances (D1 and D2, respectively, the distance from the base of the contact point of the crowns to the implant-bone contact and distance from the bottom of the crown contact point to the bone crest in the adjacent tooth) and that the papilla deficit between two implants was significantly less between implant-tooth. Furthermore, it has been shown that when the vertical distance between the gap and the implant is less than 6 mm, the papilla is always present. It was also revealed that the incidence of the papilla between two zirconium dioxide implants is higher compared to the implant-tooth group (12).

According to Chu *et al.*, there is a significant association between the papilla deficit and the height of the contact point. The ideal ratio of the height of the contact point to the height of the clinical crown is approximately 40%. More precisely, low contact points were associated with full papillae, while high contact points were associated with a deficit of papillae (30).

According to De la Rosa *et al.* and Staubli *et al.*, cementing is a risk factor for progressive marginal bone loss (31-32). According to Rodríguez *et al.* (9), the cementation is not a negative impact factor for radiographic bone loss but seems to be related. Nowzari *et al.*, Derks *et al.* and, Puisys *et al.* suggested bone and gingival biotype, systemic conditions, 3D positioning of implants, surface implantation, smoking, or a history of periodontal disease, which may have more influence on crestal bone stability (33-35).

Lower contact points in patients with a thick scalloped gingival biotype with rectangular crowns may appear more favorable than higher contact points in patients with a thin scalloped gingival biotype with triangular crown shapes and full-height papillae. Therefore, low contact points do not always result in an unpleasant aesthetic result. These findings reveal that, in implant, implant, and implant situations, weak contact points can reduce papilla deficit in some cases.

According to Kniha *et al.* there is a weak correlation between the papillary filling and the gingival biotype around the zirconium dioxide implants (11). According to Kan *et al.*, there are larger dimensions of the peri-implant mucosa in the presence of a thick biotype compared to a thin biotype (36). According to Eger *et al.*, the gingival thickness is associated with the depth of the periodontal pockets (37). But, on the other hand, Cabello *et al.* did not find a relationship between gingival biotype and soft tissue alteration (38). Different authors Kan *et al.*, Schropp *et al.*, Palmer *et al.* and Chu *et al.* have described that the distance from the alveolar crest of the tooth adjacent to the implant to the point of contact of the crowns is the main factor that affects the height of the papilla and the margin of the soft tissue (36,39-41).

Good aesthetic results and minimal complications have been demonstrated in restorations with immediate implants in the aesthetic area. This seems contradictory to the data obtained in a previous review by Chen *et al.* that showed the recession of the mid-facial mucosa after immediate implant placement (42). The results obtained by Kniha *et al.* seem to agree with the results obtained in the review by Shi *et al.*, which states that there is no significant difference in aesthetic success to peri-implant soft tissue between immediate implant loading and delays loading (16,43). There were no significant differences in the median papilla deficit between immediate loading and delayed loading implants.

-Survival rate and success of zirconium dioxide implants compared to conventional titanium implants.

On the other hand, in terms of survival, in the studies of our systematic review, zirconium dioxide implants have a high survival and success rate, which varies from 50% to 100%. So we can deduce that zirconium dioxide implants have good clinical results in the case of unit absences. An important factor, according to Morton *et al.* and Kim *et al.* in the success of zirconium dioxide implants it seems to be the primary stability, whereas, according to Schneider *et al.*, unevenly distributed occlusal contacts can contribute to implant loss (44-45).

-Limitations of the present study

More clinical studies are required to be carried out to identify all relevant biological and technical factors with impact on implant success and patient satisfaction. Most of the articles have data related to one piece ceramic implants as two piece ceramic implants are new in the market being introduced a few years back.

## Conclusions

Despite the limitations of the present study, we can conclude that:

1. Zirconium dioxide implants have shown improved aesthetic benefits in the indexes of pink and white esthetic compared to conventional titanium implants.

2. Zirconium dioxide implants have other clinical benefits compared to conventional titanium implants, showing less plaque accumulation and, therefore, less inflammation around the peri-implant mucosa. Zirconium dioxide implants have shown a lower probing depth and an ideal papilla crown proportion with increase in papillary height.

3. Considering short term studies, zirconium dioxide implants have survival rates and success rates comparable to those obtained with the conventional titanium implants.
